# Comparison of outcomes according to fixation technique following the modified Ludloff osteotomy for hallux valgus in patients with rheumatoid arthritis

**DOI:** 10.1186/s12891-017-1729-4

**Published:** 2017-08-25

**Authors:** Young-Hoon Jo, Ki-Chul Park, Young-Sik Song, Il-Hoon Sung

**Affiliations:** 1 Department of Orthopaedic Surgery, Hanyang University College of Medicie, 222-1 Wangsimni-ro, Seongdong-gu, Seoul, 04763 Republic of Korea; 2 Department of Orthopaedic Surgery, Hanyang University Guri Hospital, 153 Kyoungchun-ro, Guri-si, Gyeonggi–do 11923 Republic of Korea

**Keywords:** Rheumatoid arthritis, Hallux valgus, Metatarsal osteotomy, Fixation stability

## Abstract

**Background:**

Clinical and radiological outcomes including fixation stability of osteotomy site were compared in rheumatoid arthritis (RA) patients who underwent modified Ludloff osteotomy to correct hallux valgus with osteotomy site fixation using two screws versus those who underwent additional fixation using a plate.

**Methods:**

The fixation technique performed with two screws was used to fix the osteotomy sites following modified Ludloff osteotomy in 15 patients (15 feet, Group S), while the augmented plate fixation technique was used in 14 patients (16 feet, Group P). Surgical outcomes were analysed using the American Orthopedic Foot and Ankle Society (AOFAS) scores, and radiologic parameters measured before surgery and during follow-up examinations. To evaluate the stability of each osteotomy site fixation technique, the 1–2 inter-metatarsal angle (IMA) and angle of the altered margin of the lateral cortex (AMLC) were measured immediately and 6 weeks after surgery, and variations in the angles were compared. In addition, bone mineral density (BMD) values were compared between patients with correction loss at the osteotomy site and those with no loss of correction.

**Results:**

No significant differences between groups were found for total AOFAS scores before surgery and at the final follow-up. However, significant differences were observed in the 1–2 IMA, beginning at 6 weeks postoperatively and continuing through the final follow-up. The 1–2 IMA and angle of AMLC measured immediately after and 6 weeks after surgery showed significantly greater variation in Group S than in Group P. In Group S, patients with correction loss (5 feet) at osteotomy site showed significantly lower BMD values than those with no loss of correction (10 feet). Despite the lower BMD values of patients in Group P than in Group S, a loss of correction did not occur in these patients.

**Conclusions:**

Correction loss occurred at the osteotomy site within 6 weeks postoperatively in patients who underwent fixation using only the two-screw fixation technique following modified Ludloff osteotomy; such loss could be reduced using the augmented plate fixation technique even in patients with osteoporosis.

## Background

Rheumatoid arthritis (RA) is often accompanied by forefoot deformities, among which the most frequently occurring are hallux valgus (HV) and the hammer toe and claw toe deformities of the lesser toes [1, 2]. Approximately 20–40% of patients with RA undergo surgery because of forefoot deformities and pain. Traditionally, metatarsophalangeal (MTP) joint fusion and resection arthroplasty are performed as HV surgeries in patients with RA [1, 2]. However, in line with recent developments in RA drug treatments, many surgeons have conducted joint-preserving surgeries in patients showing minimal erosion of the MTP joint and have reported satisfactory results [3–5].

Various surgical treatments have been introduced for preserving the MTP joint. Generally, first metatarsal proximal osteotomy combined with distal soft-tissue realignment is performed for patients with symptomatic HV in whom 1–2 inter-metatarsal angles (1–2 IMA) exceed 15° [6]. The Ludloff osteotomy, a surgical method that produces relatively high biomechanical stability compared to that produced by other first metatarsal proximal osteotomy procedures, is reported to have satisfactory clinical results and is preferred by many surgeons [6–10]. We have used an osteotomy method, which is a modified version of the Ludloff osteotomy for correction of serious deformities in patients with HV; however, we observed loss of correction at the osteotomy sites in several RA patients when using only two screws for fixation. To reduce this correction loss, we next attempted additional fixation using a plate on the medial side along with two screws at the osteotomy site following the modified Ludloff osteotomy to correct HV in patients with RA.

The purpose of the present study was to compare the clinical and radiological outcomes of HV surgery in patients with RA who underwent osteotomy site fixation using only two screws after the modified Ludloff osteotomy and those who underwent additional fixation using a plate.

## Methods

### Subjects

This retrospective case-controlled study was conducted after approval from the Institutional Review Board at Hanyang University Hospital (HYUH 2016–03-008), and all patients provided their informed consent. Ninety-two patients with RA (108 feet) underwent HV surgery at the Hanyang University Medical Centre between March 2005 and December 2014. Excluding the patients who underwent first MTP joint fusion, first MTP joint resection arthroplasty, or first metatarsal distal osteotomy, 29 patients (31 feet) with at least 1 year of follow-up after modified Ludloff osteotomy were included as subjects.

In the present study, we performed joint-preserving HV surgery only in patients showing minimal erosion of the first MTP joints (below Larsen grade 2) [11]. Moreover, we performed the modified Ludloff osteotomy when moderate-to-severe HV deformities were present. We used to fix the modified Ludloff osteotomy sites using two Barouk screws (Depuy International, Leeds, England) until December 2010. However, after observing a loss of correction in several RA patients, since January 2011, we implemented the augmented plate fixation technique, which involves the use of a metal plate in addition to the two Barouk screws for fixation of osteotomy sites.

During HV surgery for patients with RA, the fixation technique performed with two Barouk screws was used to fix the osteotomy sites in 15 patients (15 feet, Group S), while the augmented plate fixation technique was used in 14 patients (16 feet, Group P). The baseline data from these two groups are summarised in Table 1.

**Table 1:** Comparison of Demographic Data between Group S and Group P

	Group S	Group P	*p* value
Patients (number of feet)	15 (15)	14 (16)	
Age, years [mean (range)]	51.4 (30–65)	57.4 (45–69)	0.064
Sex	All female	All female	1.000
Bone mass index, kg/m^2^ [mean (SD)]	22.9 (2.2)	22.7 (3.5)	0.843
Side [right: left]	7: 8	5: 11	0.379
Follow up duration, months [mean (range)]	33.3 (12–84)	22.2 (12–50)	0.080
Duration of rheumatoid arthritis, years [mean (range)]	10.5 (3–24)	13.4 (5–27)	0.232
Preoperative CRP, mg/dl [mean(range)]	0.2 (0–1.7)	0.3 (0–1.5)	0.572
Preoperative ESR, mm/h [mean(range)]	21.9 (2–52)	27.3 (2–66)	0.379
Preoperative DAS28-ESR [mean(range)]	3.57 (2.39–5.88)	3.63 (2.45–5.00)	0.834
Preoperative Larsen grade of the first MTP joint [0:1:2:3:4:5]	3:4:8:0:0:0	4:5:7:0:0:0	1.000
Akin osteotomy performed [n (%)]	11 (73)	14 (88)	0.394
Lesser toe procedures [n (%)]			0.376
Resection arthroplasty Weil osteotomy	5 (33) 1 (6)	6 (37) 4 (25)	
Operation time, minutes [mean (SD)]	137 (41.6)	164 (42.5)	0.163
DMAA, ° [mean(range)]	13.4 (3.4–28.8)	14.3 (4.8–25.4)	0.545
Preoperative femoral neck BMD, g/cm^2^ [mean (SD)]	0.672 (0.117)	0.592 (0.079)	0.033*
Preoperative femoral neck T-score [mean (SD)]	−1.2 (1.1)	−2.0 (0.72)	0.036*

*Significant difference

*CRP* C-reactive protein, *ESR* Erythrocyte sedimentation rate

*DAS28-ESR* Disease Activity Score using 28 joint counts based on ESR, *MTP* metatarsophalangeal

*DMAA* Distal metatarsal articular angle, *BMD* Bone mineral density

### Surgical technique and post-operative protocol

The surgery was performed by one senior surgeon (IHS). A longitudinal incision (approximately 7 cm in length) was made on the medial side of the bunion and the shaft of the first metatarsal in order to remove the bunion using a saw in a direction parallel to the longitudinal axis of the first metatarsal shaft. We used a modified version of Ludloff osteotomy, which involved the following. A longitudinal oblique osteotomy was performed from the plantar-distal aspect of the first metatarsal shaft immediately proximal to the sesamoid complex and directed to the dorsal-proximal aspect. At the end of osteotomy, a vertical step-cut osteotomy was made on the dorsal cortex, which gave additional stability and prevented excessive shortening with its buttressing effect to the distal fragment. After laterally transposing the distal fractured fragments of the first metatarsal to obtain 1–2 IMA to approximately 5°, the osteotomy site was fixed using two Barouk screws. Starting January 2011, the osteotomy site was augmented in RA patients using an LCP Compact Hand 2.0-mm or 2.4-mm (Synthes, Oberdorf, Switzerland) on the medial side, in addition to the Barouk screws.

The hallux valgus angle (HVA) was corrected by making an additional incision (approximately 2 cm in length) in the first web space, and distal soft-tissue realignment was subsequently conducted. If the HVA correction was insufficient or if excessive tension was applied on the distal soft tissues, an additional Akin osteotomy was performed as needed. Lesser toe deformities were corrected selectively by resection arthroplasty or Weil osteotomy based on the severity and flexibility of the deformity and the extent of MTP joint erosion.

Immediately after surgery, non-weight-bearing plain radiographs were obtained, and all patients were subsequently allowed to place weight on their heels while wearing postoperative shoes beginning 1 week after surgery. At 3 weeks after surgery, sutures were removed while the patient was in the outpatient clinic, and full weight-bearing was allowed when wearing regular shoes at 6 weeks postoperatively. To maintain the corrected hallux position, patients were advised to keep their dressing bandage in place for 6 weeks after surgery.

### Patient evaluation and follow-up

All patients who underwent HV surgery were evaluated preoperatively and at 6 weeks, 3 months, 6 months, and 12 months postoperatively. These visits were followed by an annual check-up to measure the American Orthopaedic Foot and Ankle Society (AOFAS) hallux metatarsophalangeal-interphalangeal score and patients’ subjective satisfaction as well as to obtain weight-bearing plain radiographs.

For clinical evaluation, preoperative and final follow-up AOFAS scores of the two groups were compared. When comparing the AOFAS scores, we analysed the total scores as well as the scores for each subscale, which included pain (40 points), function (45 points), and alignment (15 points). In addition to the AOFAS scores, the level of patients’ subjective satisfaction was recorded at the final follow-up for comparison and categorised into “excellent”, “good”, “fair”, or “poor” [12].

For radiological evaluation, HVA, 1–2 IMA, and sesamoid position were compared between the two groups preoperatively, immediately after surgery, 6 weeks and 6 months postoperatively, and at the final follow-up. The HVA was measured as the angle between the line connecting the centre of first metatarsal’s proximal articular surface to the centre of the first metatarsal head and the line connecting the midpoints of proximal and distal articular surfaces of the proximal phalanx [13]. The 1–2 IMA was measured as the angle between the line of first metatarsal bone and the line bisecting the second metatarsal shaft. The sesamoid positions were assessed using the method described by Hardy and Clapham; they were graded from I to VII based on the positional relationship between the medial sesamoid and the longitudinal axis of first metatarsal (I, most medial; VII, most lateral) [14].

**Fig. 1:**
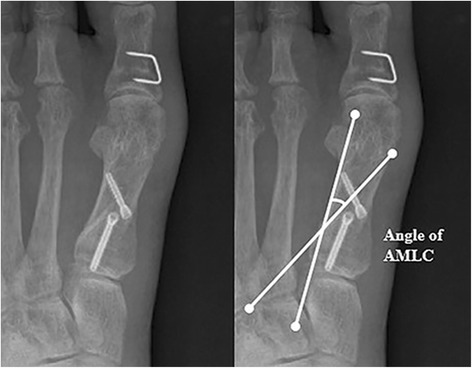
The angle of altered margin of lateral cortex (AMLC) was measured as the angle between the line of proximal lateral cortex and the line of distal lateral cortex of the first metatarsal bone

To evaluate the degree of fixation stability at the osteotomy site for each fixation technique, the absolute values of angle variations were compared by measuring the angle of altered margin of the lateral cortex (AMLC) and 1–2 IMA, immediately and 6 weeks after surgery. The angle of AMLC was defined as the angle between the proximal and distal lateral cortices on the osteotomy site (Fig. 1), while a loss of correction was defined when the 1–2 IMA and angle of AMLC measured 6 weeks after surgery changed by more than 5° from those measured immediately after surgery. We could not measure the angle of AMLC after 6 weeks following surgery because the angle was not accurately identified due to united osteotomy sites. Furthermore, the HVA recurrence rates of the two groups at the final follow-up were compared. Recurrence was defined as HVA ≥20° [15].

The bone mineral density (BMD) was compared between patients with correction loss at the osteotomy site and those with no loss of correction. Hanyang University Medical Centre has a special institution for RA patients (Hanyang University Hospital for Rheumatic Disease), and most RA patients who visit the department of orthopaedic surgery for forefoot surgery were transferred from this institution. Areal BMD measurement was annually performed for the lumbar vertebra and femoral neck in most RA patients using dual energy X-ray absorptiometry (DXA) (Discovery QDR, Hologic, Bedford, MA, USA) for the evaluation of osteoporosis at the department of rheumatology. Therefore, most RA patients who underwent forefoot surgery at our institution had DXA performed within 1 year. Of the preoperative measurements of femoral neck BMD from both sides, we used the lower values for analysis.

For radiographic measurements, two orthopaedic surgeons (YHJ, YSS) who did not participate in the surgeries, measured radiological parameters using the PACS π view star (Infinitt, Seoul, Korea) digital measurement program at two sessions with a 2-week interval in between, and mean values of the measurements were used for analysis. To evaluate intra- and inter-observer concordance, intra-class coefficients (ICC) and Kappa coefficients, respectively, for HVA, 1–2 IMA, sesamoid position, and angle of AMLC were calculated.

In addition to the clinical and radiological outcomes, complications, including implant irritation, infection, non-union, deep vein thrombosis, first MTP joint arthrosis, and delayed wound healing were compared between the two groups.

### Statistical analysis

SPSS software (version 18.0; SPSS, Inc., Chicago, Illinois) was used for statistical analysis. Continuous variables were compared using the Student’s t-test or the Mann-Whitney U-test, depending on whether they were normally distributed. Paired t-tests or Wilcoxon signed rank tests were used to compare AOFAS scores, HVA, 1–2 IMA, and sesamoid position before surgery and at the final follow-up. The Kolmogorov-Smirnov test and Shapiro-Wilk test were used to evaluate normal distribution of the data. The chi-squared test or Fisher’s exact test was used to compare dichotomous data. To compare differences in the effect of time on postoperative outcomes between the two groups, a mixed model analysis was used. The mixed model analysis is a statistical method used for analysing the differences in repeated measurements between groups. The HVA, 1–2 IMA, and sesamoid position were used in this analysis, measured repeatedly after surgery. Differences were deemed statistically significant when *p* < 0.05.

## Results

### Demographic data

No statistically significant difference was found between the two groups in the following categories: age (*p* = 0.069), sex (*p* = 1.000), body mass index (*p* = 0.843), side (*p* = 0.379), postoperative outpatient follow-up duration (*p* = 0.080), duration of RA (*p* = 0.226), preoperative C-reactive protein levels (*p* = 0.572), preoperative erythrocyte sedimentation rate (ESR) levels (*p* = 0.358), preoperative Disease Activity Score using 28 joint counts based on ESR (DAS28-ESR) (*p* = 0.834), preoperative Larsen grade of the first MTP joint (*p* = 1.000), whether Akin osteotomy was performed (*p* = 0.394), whether a lesser toe procedure was performed (*p* = 0.376), operation time (*p* = 0.163), and distal metatarsal articular angle (DMAA) (*p* = 0.545). However, femoral neck BMD and T-score were significantly lower in Group P than in Group S (*p* = 0.043, *p* = 0.039) (Table 1).

With respect to RA medications used by the patients in Group S (15 patients) and Group P (14 patients), statistically significant differences were not detected (*p* = 0.750): eight patients from Group S (53%) and nine patients from Group P (64%) were prescribed methotrexate only; three patients from Group S (20%) and four patients from Group P (29%) were prescribed more than two disease-modifying anti-rheumatic drugs (DMARDs), such as methotrexate, sulfasalazine, and hydroxychloroquine; and three patients from Group S (20%) and one patient from Group P (7%) were prescribed both methotrexate and etanercept. One patient from Group S was not prescribed any RA medication within 1 year prior to the surgery.

### Clinical outcomes

AOFAS scores measured before surgery and at the final follow-up are summarised in Table 2. The total AOFAS scores measured at the final follow-up were significantly better in both groups compared to those obtained before surgery (all *p* < 0.001). The two groups did not differ significantly in terms of the preoperative and final follow-up total AOFAS scores (*p* = 0.861; *p* = 0.096). Similarly, significant differences were not observed in the pain and function subscales of the AOFAS scores between the two groups. However, Group P scored significantly higher in the alignment subscale at the final follow-up (*p* = 0.004).

**Table 2:** Comparison of AOFAS Scores between Group S and Group P

	Group S	Group P	*p* value
Preoperative total score [mean (SD)]	45.9 (12.7)	46.8 (12.4)	0.861
Pain subscale	18.7 (8.3)	20.0 (8.9)	0.626
Function subscale	24.8 (5.7)	24.8 (5.8)	0.770
Alignment subscale	2.1 (3.7)	1.5 (3.2)	0.711
Final follow-up total score [mean (SD)]	74.7 (17.9)	83.7 (10.4)	0.096
Pain subscale	31.3 (10.6)	32.5 (5.7)	0.892
Function subscale	36.6 (6.2)	38.8 (4.2)	0.281
Alignment subscale	6.8 (4.9)	12.4 (3.5)	0.004*
*p-value* ^*a*^	< 0.001*	< 0.001*	

*Significant difference

*p*-values^a^ were estimated by comparing preoperative total score values and total score values at the final follow-up

Subjective patient satisfaction levels measured at the final follow-up in the two groups were as follows: five Group S patients (33%) and eight Group P patients (57%) indicated that their satisfaction levels were “excellent”; five Group S patients (33%) and four Group P patients (29%) reported “good” satisfaction levels; three Group S patients (20%) and two Group P patients (14%) responded with “fair” satisfaction levels; and only two patients (13%), both of whom were in Group S, experienced a “poor” level of satisfaction.

### Radiologic outcomes

The intra-observer reliabilities for HVA, 1–2 IMA, sesamoid position, and AMLC angle showed an ICC of 0.87 (95% confidence interval [CI], 0.82–0.92), ICC of 0.92 (95% CI. 0.86–0.98), kappa coefficient of 0.82 (95% CI, 0.74–0.90), and ICC of 0.80 (95% CI, 0.70–0.88), respectively, demonstrating outstanding reliability. Inter-observer reliability for these same variables showed an ICC of 0.83 (95% CI, 0.75–0.91), ICC of 0.88 (95% CI, 0.82–0.94), kappa coefficient of 0.76 (95% CI, 0.69–0.82), and ICC of 0.72 (95% CI, 0.64–0.80), respectively, further confirming the reproducibility of results.

**Fig. 2:**
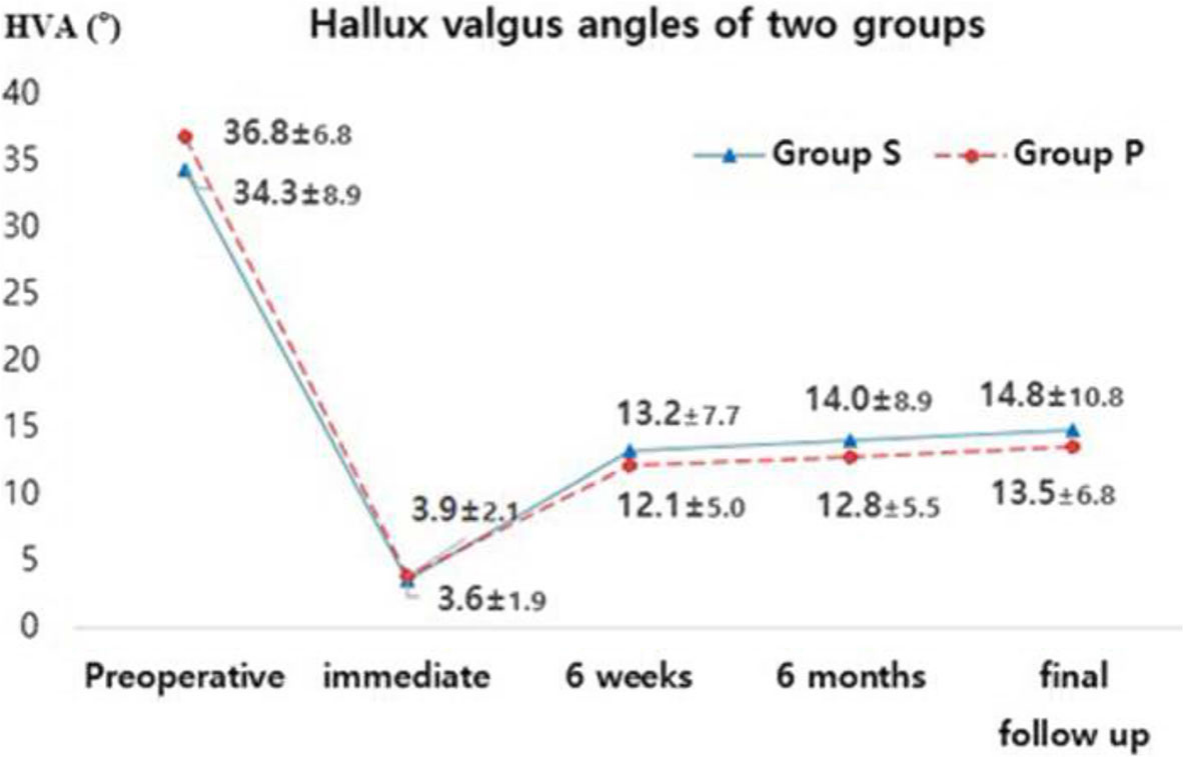
Graph showing changes in the hallux valgus angle over time in both groups (all *p* > 0.05)

**Fig. 3:**
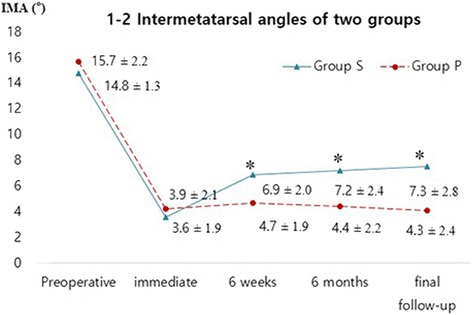
Graph showing changes in the 1–2 inter-metatarsal angle over time in both groups (*p < 0.125, significance level was corrected to 0.0125 with the Bonferroni method)

**Fig. 4:**
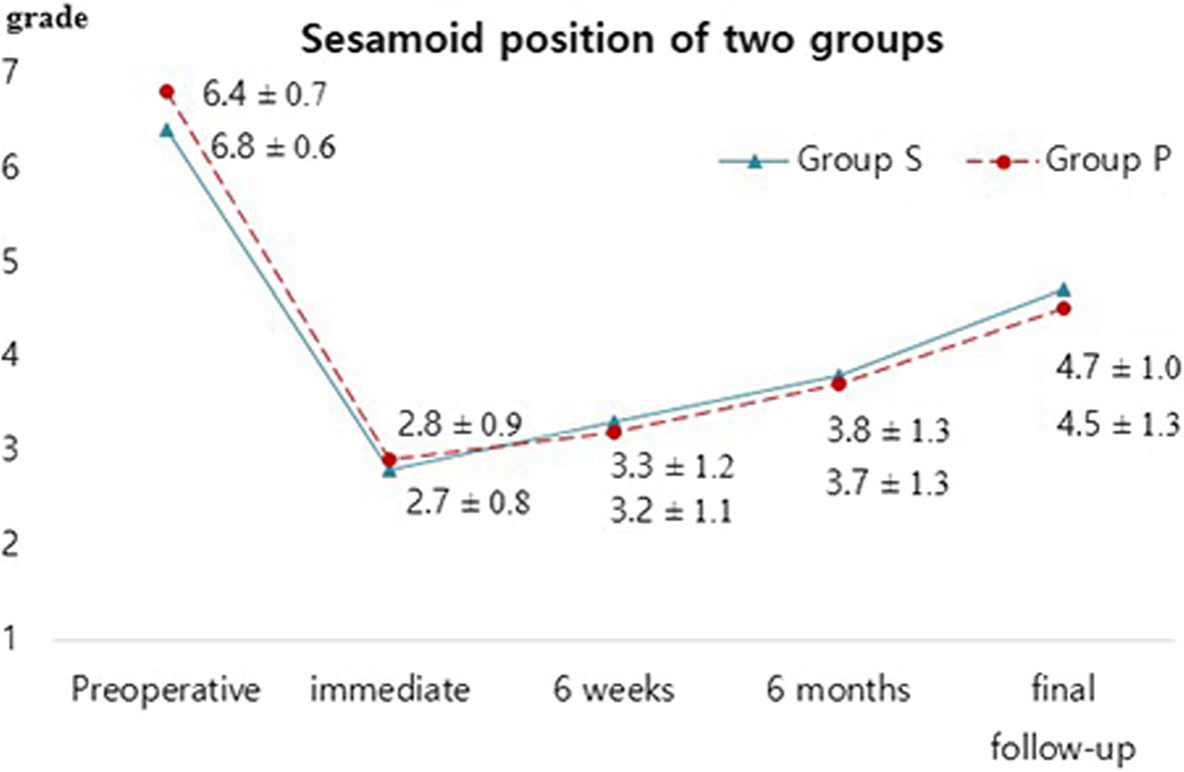
Graph showing changes in the medial sesamoid position over time in both groups (all *p* > 0.05)

Changes over time in the HVA, 1–2 IMA, and sesamoid position in Group S and Group P are illustrated in Figs. 2, 3, and 4. HVA, 1–2 IMA, and sesamoid position significantly improved in the two groups at final follow-up compared to those measured before surgery (all *p* < 0.001). HVA (*p* = 0.456, *p* = 0.727) and sesamoid position (*p* = 0.259, *p* = 0.488) measured before surgery and at the final follow-up were not significantly different between the two groups (Table 3). Regarding 1–2 IMA measured before surgery, no significant difference was observed between the two groups (*p* = 0.066); however, significant differences were found at the final follow-up (*p* = 0.001). Using the mixed model analysis, no difference by time interaction was found for postoperative HVA (*p* = 0.662) and sesamoid position (*p* = 0.824); however, a significant difference by time interaction was found for postoperative 1–2 IMA (*p* = 0.011) between the two groups (Figs. 2, 3, and 4). The pairwise comparisons to examine differences in 1–2 IMA between groups at each of the follow-up points where 1–2 IMA measurement was immediately performed after surgery were not significantly different between the groups (*p* = 0.748). However, at six weeks after surgery, the mean 1–2 IMA of Group S was significantly higher than that of Group P, and that difference was maintained until the final follow-up (significance level was corrected to 0.0125 with the Bonferroni method; *p* = 0.004 at 6 weeks, *p* = 0.002 at 6 months, and *p* = 0.004 at the final follow-up).

**Table 3:** Comparison of Radiologic Outcomes between Group S and Group P

	Group S	Group P	*p* value
Hallux valgus angle, ° [mean (SD)]			
Preoperative	34.3 (8.9)	36.8 (6.8)	0.380
At the final follow-up	14.8 (10.8)	13.5 (6.8)	0.922
*p-value* ^*a*^	< 0.001*	< 0.001*	
1–2 Intermetatarsal angle, ° [mean (SD)]			
Preoperative	14.8 (1.3)	15.7 (2.2)	0.066
At the final follow-up	7.3 (2.8)	4.3 (2.4)	0.004*
*p-value* ^*a*^	< 0.001*	< 0.001*	
Sesamiod postion grade, grade 1 to 7 [mean(SD)]			
Preoperative	6.4 (0.7)	6.8 (0.6)	0.202
At the final follow-up	4.7 (1.0)	4.5 (1.3)	0.545
*p-value* ^*a*^	< 0.001*	< 0.001*	

*Significant difference

*p*-values^a^ were estimated by comparing preoperative values and values at the final follow-up

**Fig. 5:**
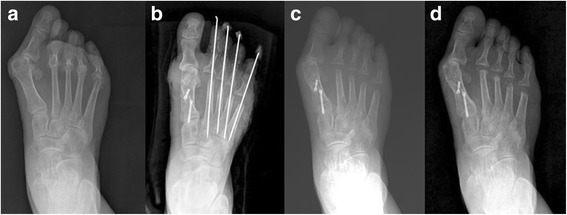
A case with correction loss in Group S: **a** A preoperative radiograph of a 49-year-old woman with rheumatoid arthritis. **b** A postoperative radiograph taken immediately after surgery. **c** The 1–2 inter-metatarsal angle and altered margin of lateral cortex measured immediately and 6 weeks after surgery changed by more than 5°, as shown on plain radiography. **d** Radiograph taken at 32 months following surgery

**Fig. 6:**
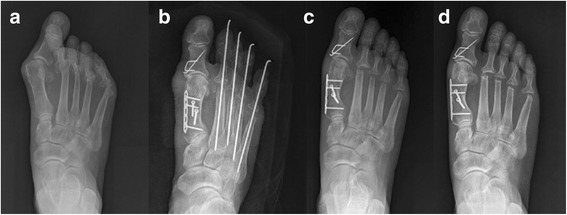
A case with plate augmentation: **a** A preoperative radiograph of a 61-year-old woman with rheumatoid arthritis. **b** A postoperative radiograph taken immediately after surgery. **c** The 1–2 inter-metatarsal angle and altered margin of lateral cortex changed by less than 5°. **d** Radiograph taken at 24 months following surgery

An analysis of the fixation stability of osteotomy sites showed that mean variations in the 1–2 IMA and angle of AMLC measured immediately and 6 weeks after surgery in Group S were 3.3° and 4.7°, respectively, and 0.9° and 2.1°, respectively, in Group P (*p* = 0.001, *p* = 0.024). A loss of correction was observed in five feet in Group S (33%) and 0 feet in Group P (0%) (*p* = 0.018) (Figs. 5, 6). There were no significant differences in HVA, 1–2 IMA, and sesamoid position measured before and immediately after surgery between the correction loss and correction maintenance subgroups within Group S (Table 4). The five feet in correction loss subgroup within Group S showed significantly lower BMD values compared to those for the remaining 10 feet in which no loss of correction occurred (*p* = 0.023) (Table 5). There were no significant differences in BMD values between Group P and the correction loss subgroup within Group S, and no patients in Group P showed correction loss (Table 5).

**Table 4:** Comparison of parameters between correction maintenance and correction loss subgroups within Group S

	Correction maintenance group (*n* = 10)	Correction loss group (*n* = 5)	*p* value
Hallux valgus angle, ° [mean (SD)]			
Preoperative	32.1 (9.5)	38.6 (6.3)	0.206
Immediate postoperative	3.2 (2.4)	5.0 (4.8)	0.310
1–2 Intermetatarsal angle, ° [mean (SD)]			
Preoperative	14.4 (1.4)	15.4 (0.8)	0.129
Immediate postoperative	4.0 (2.2)	3.0 (0.9)	0.679
Sesamiod position grade, grade 1 to 7 [mean(SD)]			
Preoperative	6.2 (0.8)	6.8 (0.4)	0.206
Immediate postoperative	2.8 (0.8)	2.6 (0.9)	0.679

**Table 5:** Bone Mineral Density (BMD) Analysis

	Preoperative femoral neck BMD, g/cm^2^ [mean (SD)]	Preoperative femoral neck T-score [mean (SD)]
Group P	0.592 (0.079)	−2.0 (0.72)
Group S	0.672 (0.117)	−1.2 (1.11)
Correction Maintenance (*n* = 10)	0.720 (0.114)	−0.8 (1.01)
Correction Loss (*n* = 5)	0.577 (0.046)	−2.1 (0.43)
*p*-value	0.028*	0.028*
*p-value* ^*a*^	0.905	0.905

*Significant difference

*p*-values were estimated by comparing the correction maintenance and correction loss subgroups within Group S

*p*-values^a^ were estimated by comparing Group P with the correction loss subgroup within Group S

Furthermore, HVA recurrence (HVA of ≥20°) was detected at the final follow-up in five feet (33%) in Group S and four feet (25%) in Group P, but these differences were not statistically significant (*p* = 0.704).

### Complications

Two patients (13%) in Group S reported persistent symptoms of bunion pain and symptomatic HV, but no such HV symptoms were reported in Group P. Implant irritation caused by plates occurred in five feet (31%) in Group P, and consequently, one patient underwent surgery for implant removal. Other complications such as non-union, infection, deep vein thrombosis, and first MTP joint arthrosis were not observed. Both groups had one patient each who showed delayed wound healing as the normal healing process was not achieved within 3 weeks, but these wounds healed after conservative treatment.

## Discussion

With recent developments in DMARDs and biologic agents, RA disease activity is well controlled, and the progression of joint destruction can be prevented [16, 17]. In RA patients undergoing HV surgery, joint-preserving surgery can be performed on first MTP joints that show minimal erosion. Various studies have recently reported joint-preserving surgery for correcting HV in patients with RA, with most of them resulting in satisfactory clinical and radiological outcomes [1, 3–5, 18].

Compromised bone stock is one of the factors to be considered in joint-preserving surgery in patients with RA [19]. Osteoporosis often occurs in these patients as a result of long-term steroid use, disability-associated immobility, and excessive osteoclast activation. In addition, unlike postmenopausal osteoporosis, it usually causes bone loss in the peripheral cortical bones, such as metatarsal bones [20]. In general, screws can sufficiently maintain osteotomy site fixation in HV surgery after modified Ludloff osteotomy. However, these could be insufficient in patients with RA because of their osteoporotic bones, thereby resulting in loss of correction at the osteotomy site [21, 22].

In an attempt to reduce the loss of correction at the osteotomy site following HV surgery in patients with RA, we implemented the augmented plate fixation technique for fixation of the modified Ludloff osteotomy site. Although periarticular bone loss, such as in the hand and foot is associated with generalized bone loss in RA patients [23], the progression of periarticular bone loss is known to occur earlier than hip or spine bone loss [24, 25]. Moreover, since RA patients have reduced bone quality as well as bone quantity [26, 27], we have been performing augmented plate fixation in all RA patients since 2011, regardless of their hip BMD values. The radiological results from this study showed that the two groups differed significantly in terms of 1–2 IMA measured at 6 weeks postoperatively, whereas no difference was observed immediately after surgery. These differences may be attributed to the loss of correction that occurred in several Group S patients due to insufficient fixation with screws alone. Indeed, patients who experienced loss of correction showed significantly low BMD as well (Table 5).

Most cases of correction loss at osteotomy sites occur within 6 weeks after surgery [28]. We compared the 1–2 IMA and angle of AMLC immediately and 6 weeks after surgery to define loss of correction as the point when the two parameters changed by more than 5°. Plain radiographs were obtained in non-weight-bearing conditions immediately after surgery and in weight-bearing conditions 6 weeks later. According to previous studies, radiologic parameters change depending on weight-bearing conditions [29, 30]. In particular, Tanaka et al. reported that the 1–2 IMA could increase by up to 4.7° in weight-bearing conditions [29]. We therefore, defined loss of correction as a change of more than 5° as our standard considering that the study by Tanaka et al. and the 1–2 IMA values showed excellent intra- and inter-observer reliability [31]. Furthermore, we introduced the angle of AMLC as another parameter. The angle of AMLC, which is the angle between the proximal and distal lateral cortices on osteotomy sites (Fig. 1), remains constant regardless of weight-bearing conditions and allows a good judgment on loss of correction at osteotomy sites. The intra- and inter-observer reliability for the angle of ALMC is not known, but the results in our study exhibited a good reliability, with an ICC >0.7.

Various studies have shown that plate fixation is biomechanically superior to screw fixation after proximal metatarsal osteotomy [21, 32]. However, while acknowledging the biomechanical superiority of plate fixation, Park et al. [33] reported that radiologic outcomes were worse after plate fixation than after K-wire fixation since loss of correction occurred at the osteotomy sites during the plate fixation process conducted after proximal metatarsal osteotomy. Instead of using plate fixation alone, we implemented an additional plate augmentation method; two screws were first fixed on the modified Ludloff osteotomy site, and a plate was subsequently augmented on the medial side to prevent correction loss at the site during plate fixation. Although Group P did not show a significant difference in BMD values against the correction loss subgroup within Group S (Table 5), a loss of correction did not occur in these patients because of potential high biomechanical stability resulting from the augmented plate fixation technique. Nonetheless, further biomechanical studies are needed to investigate this aspect.

No significant difference was found between the two groups in HVA, sesamoid position, and the recurrence rate of HVA at the final follow-up. In Group P, no patients had loss of correction at the osteotomy site, but there were four patients (25%) with HVA ≥20° at the final follow-up. Various factors, including an insufficient release of the soft tissues, increased DMAA, and under-correction or loss of correction of the first metatarsal, are known to be involved in HVA recurrence [34]. Significant differences between the two groups seem to be absent because other factors, such as soft-tissue incompetence, are involved in HVA recurrence, in addition to loss of correction of the metatarsal. However, three out of five Group S patients that experienced recurrence showed loss of correction at the osteotomy sites, and two of these reported persistent bunion pain and transfer metatarsalgia.

Park et al. [33] reported that they performed the plating technique in 21 cases after proximal chevron osteotomy and performed plate removal in 12 cases because of irritation (57%). In the present study, five patients (31%) in Group P reported plate irritation, thus showing a relatively high incidence. However, four of them showed only mild symptoms, and thus, did not undergo plate removal. One patient who underwent HV surgeries on both feet underwent plate removal on the previously operated side while undergoing the operation on the other side, resulting in the disappearance of irritation symptoms and high satisfaction of the patient after plate removal.

This study has a few limitations. First, it was a retrospective study conducted on a relatively small number of patients. Therefore, it has low statistical power. Second, surgical experiences could have affected the results because of differences in the timing of surgeries. However, one senior surgeon (IHS) who has performed foot and ankle surgery since 1997 performed all operations; hence, the level of surgical skills may be less affected. Third, the follow-up duration in the two groups differed, though they were not statistically significant. Lastly, the simple radiographs obtained immediately after surgery in non-weight-bearing conditions could have resulted in errors.

## Conclusions

In this study, both fixation methods resulted in significant improvements in the AOFAS score, HVA, 1–2 IMA, and sesamoid position at the final follow-up when compared to those measured before surgery in HA patients with RA. The use of plate augmentation in addition to two screws significantly lowered correction loss at the osteotomy site, regardless of BMD values, compared to the use of the screw fixation technique alone. In conclusion, methods to enhance fixation stability at the osteotomy site must be considered before HV surgery, in accordance with the findings from bone strength assessment.

## Abbreviations

1–2 IMA: 1–2 inter-metatarsal angles
AMLC: altered margin of lateral cortex
AOFAS: American Orthopaedic Foot and Ankle Society
BMD: bone mineral density
CI: confidence interval
DMAA: distal metatarsal articular angle
DMARDs: disease-modifying anti-rheumatic drugs
ESR: erythrocyte sedimentation rate
HV: hallux valgus
HVA: hallux valgus angle
ICC: intra-class coefficients
MTP: metatarsophalangeal
RA: rheumatoid arthritis
